# A data-driven stratification framework for physical fitness in junior high school students: a composite approach based on BMI and multidimensional data

**DOI:** 10.3389/fpubh.2026.1959431

**Published:** 2026-09-28

**Authors:** Yi Fang, Juhao Hou, Shilin Wen

**Affiliations:** Capital University of Physical Education and Sports, Beijing, China

**Keywords:** adolescent health, body mass index (BMI), cluster analysis, differentiated instruction, physical fitness

## Abstract

**Objective:**

To overcome the practical challenges of implementing differentiated instruction in large physical education (PE) classes, this study develops a composite stratification framework. This exploratory framework provides a baseline for precise, individualized physical fitness interventions in adolescents.

**Methods:**

A quantitative cross-sectional design was employed. First, a questionnaire survey was administered to 125 primary and secondary school PE teachers in Beijing to evaluate their attitudes and preferences for evaluation indicators regarding differentiated instruction. Second, multidimensional physical fitness monitoring data were collected from 532 junior high school students. Following the standardization of continuous physical fitness test scores and categorization by Body Mass Index (BMI), one-way analysis of variance (ANOVA) and K-means clustering algorithms were comprehensively applied to explore the underlying latent cluster formations of students' multidimensional physical fitness characteristics.

**Results:**

The survey revealed a high theoretical acceptance of differentiated instruction among frontline teachers, yet a “knowing-doing gap” remains in large-class settings, with 65.85% of respondents prioritizing BMI as the primary stratification criterion. ANOVA confirmed significant differences in physical fitness scores across BMI groups, validating its preliminary screening value. However, it also demonstrated that relying on a single morphological indicator easily obscures the micro-heterogeneity of unbalanced physical performance. The introduction of multidimensional data clustering successfully grouped male students into 2 clusters and female students into 3 clusters, effectively controlling pedagogical stratification tiers and intra-group variance.

**Conclusion:**

The composite stratification framework—integrating initial BMI screening with multidimensional physical fitness data clustering—serves as an exploratory framework for identifying heterogeneous fitness profiles. Because this study identifies statistical subgroups cross-sectionally without prospective interventions or safety monitoring, it does not constitute a validated educational strategy. Its practical pedagogical effectiveness, longitudinal stability, and safety require rigorous external validation studies.

## 1 Introduction

In recent years, the continuous decline in the physical fitness levels of Chinese adolescents, alongside the rapid escalation of overweight and obesity rates, has emerged as a major public health concern closely monitored by society (1). Numerous studies have confirmed that insufficient physical activity is a critical factor leading to health issues such as physical decline, obesity, and myopia among adolescents. Meanwhile, structural factors, including excessive academic burdens and an exam-oriented education system, are significant barriers restricting students' participation in physical exercise (2). Globally, a lack of physical activity has become an independent risk factor for cardiovascular and metabolic diseases, with up to 80% of adolescents failing to meet the daily moderate-to-vigorous physical activity standards recommended by the World Health Organization (WHO) (3). In response to this severe situation, the national government has placed high importance on students' physical health, successively introducing a series of policies to guide physical health interventions for youth. However, how to effectively implement these policies in specific classroom teaching practices remains an urgent practical problem to be solved.

In physical education (PE) practice, there are significant individual differences in students' foundational physical fitness, athletic abilities, and health levels. The theory of Differentiated Instruction, initially proposed by Tomlinson, explicitly advocates for accommodating students' individual needs by adjusting teaching content, processes, and products (4). This theoretical framework has been widely established as a core pedagogical paradigm for addressing classroom diversity and has been repeatedly validated and applied in recent empirical research (5). As a specific implementation strategy of differentiated instruction, tiered instruction implies that all learners use the same curriculum materials, which are then modified based on content depth, learning activities, or the types of outcomes produced by students to suit their readiness levels, interests, or learning needs (5). As an effective approach to responding to individual differences, tiered instruction has garnered widespread attention in the educational field. Existing studies indicate that tiered instruction can effectively boost classroom engagement, facilitate motor skill mastery, and improve physical health (6). Furthermore, it significantly enhances motor skills and coordination, learning motivation, social interaction, and emotional wellbeing, while simultaneously optimizing teachers' professional development (7). However, the implementation of tiered instruction in practice faces multiple challenges. For instance, large class sizes make it difficult for teachers to provide meticulous guidance to each group after stratification, and limited school sports facilities struggle to simultaneously accommodate the practice needs of multiple groups (6). These dilemmas largely hinder the true realization of tiered instruction in PE classrooms. Therefore, for junior high school students who are in a critical period of physical remodeling, it is particularly vital to construct an objective stratification indicator system driven by empirical data. Such a system must be able to precisely characterize the heterogeneity of students' physical fitness while enabling low-cost, high-efficiency screening in environments with high student-teacher ratios.

Junior high school students, generally aged 13–16, are in the most dramatic and sensitive “window of opportunity” in the process of human growth and development. As explicitly stated in the World Bank's report on child and adolescent health and development (8), the ages of 10–14 mark the “pubertal growth spurt” phase, during which the physical growth rate reaches its peak since age two, serving as a critical period for remedial catch-up growth. The ages of 15–19 constitute the growth consolidation phase, representing the final opportunity for height catch-up. Therefore, students in this stage are in a crucial window for physical development. Influenced by the hormones associated with pubertal sexual maturation, physical training during this period needs to transition from purely neural adaptation to a joint neural and hormonal adaptation (9), with a focus on developing qualities such as strength, speed, and agility.

Consequently, junior high school physical education (PE) classrooms must place physical fitness training in a prominent position. While the Physical Education and Health Curriculum Standards for Compulsory Education lists elements like Body Mass Index (BMI), speed, and endurance as crucial components, relying on a single morphological indicator for pedagogical *stratification* is fundamentally flawed. BMI serves merely as an efficient, zero-cost preliminary screening tool; because it possesses extremely high inter-rater reliability (ICC ≥ 0.96) and requires no complex testing equipment, it allows teachers to quickly establish a rapid morphological baseline for the entire class (10). However, as a proxy for adiposity, BMI has well-documented limitations in adolescent populations. It cannot accurately differentiate between fat-free mass (e.g., skeletal muscle) and fat mass, nor does it account for individual variations in biological maturation and pubertal growth spurts (11). Consequently, a substantial number of students with hidden physical vulnerabilities are camouflaged under the “normal weight” label. To resolve this contradiction and advance existing differentiated instruction frameworks, PE practices must move beyond unidimensional assessments. Our proposed approach clearly distinguishes between three conceptual steps: categorization (the initial rapid risk screening using predefined BMI cutoffs), clustering (the unsupervised statistical grouping of multidimensional fitness data), and stratification (the overarching pedagogical framework combining both steps to inform differentiated instruction). In this study, BMI is prioritized strictly as a preliminary screening mechanism, which is subsequently deeply integrated with the multidimensional clustering of performance-based parameters, such as cardiorespiratory endurance and muscular strength. This sequential screening and clustering framework constitutes the theoretical starting point of our research, moving beyond basic risk assessment to construct a comprehensive, data-driven pedagogical profiling framework suitable for environments with high student-teacher ratios.

Nevertheless, current research on the stratification of students' physical health predominantly focuses on theoretical discussions or inter-group comparisons based on single indicators. Few studies construct stratification frameworks that can be directly referenced in frontline teaching based on large-scale physical fitness monitoring data. In view of this, based on a quantitative cross-sectional design, this study intends to employ cluster analysis to explore inherent structural characteristics and population stratification patterns. The objective is to construct an exploratory data-driven stratification framework for PE classrooms. Within this framework, BMI will be incorporated into the comprehensive assessment as a preliminary screening indicator. This approach retains its significant monitoring value while mitigating the risk of misjudgment associated with relying on a single indicator, thereby providing data support and methodological references for PE teachers to identify heterogeneous fitness profiles. Because this study identifies statistical subgroups cross-sectionally without prospective interventions or safety monitoring, it serves as an initial theoretical foundation rather than a validated educational strategy, and its practical pedagogical effectiveness requires rigorous external validation.

## 2 Methods

### 2.1 Questionnaire survey method

Using a convenience sampling method, frontline primary and secondary school physical education (PE) teachers in Beijing were recruited through professional municipal teaching networks *via* Wenjuanxing (an online survey platform). Prior to formal distribution, the draft questionnaire was evaluated by an expert panel comprising sports science researchers and senior frontline PE teachers to establish content validity and ensure practical pedagogical relevance. Minor adjustments were made based on their feedback. A total of 135 questionnaires were distributed, resulting in 125 valid questionnaires and an effective response rate of 92.59%. The questionnaire consisted of four sections: basic information, including demographic variables such as gender, professional title, teaching experience, and teaching city in a single-choice format; an attitude survey on the implementation of tiered instruction, comprising 10 items; an acceptance survey of stratification indicators, comprising 8 items, with both sections and measured using a five-point Likert scale (12) where options ranging from “strongly disagree” to “strongly agree” were assigned values from 1 to 5; and a ranking of stratification indicator importance, requiring teachers to rank alternative indicators by priority (13). The content related to stratification indicators was strictly designed in accordance with the physical fitness components of the athletic ability module in the Physical Education and Health Curriculum Standards for Compulsory Education (14). During data analysis, the ranking questions were quantitatively processed using a weighted scoring method, where the frequencies of each indicator ranked 1st, 2nd, and 3rd were assigned corresponding weights to calculate a composite score; a higher score indicated a higher priority perception among the teachers. All data were imported into SPSS 27.0 for statistical processing. Reliability testing on the 18 scale items from sections and yielded a Cronbach's α coefficient of 0.957, demonstrating high reliability. For content validity, all questionnaire indicators were strictly benchmarked against the aforementioned curriculum standards (15). Regarding construct validity, an exploratory factor analysis on the core scale items revealed a KMO value of 0.895 and a Bartlett's test of sphericity value of 1,566.78 (*p* < 0.001), indicating good construct validity.

To examine the structural validity of the instrument, an exploratory factor analysis (EFA) was conducted utilizing Principal Component Analysis (PCA) with Varimax rotation and Kaiser normalization. Based on the retention criterion of eigenvalues greater than 1, three common factors were extracted, collectively accounting for 66.635% of the total variance. The rotated component matrix indicated that all final items exhibited factor loadings exceeding 0.50 on their respective factors, with no apparent evidence of severe cross-loading (see Table 1).

**Table 1:** Rotated component matrix.

Item	Factor 1	Factor 2	Factor 3
I understand the foundational curriculum philosophy of the new standards regarding “focusing on individual differences and ensuring every student benefits.”	0.069	0.777	−0.014
I believe there are differences in physical fitness and sports foundations among students in the same class, which require differentiated treatment.	0.13	0.821	0.228
I believe that uniform physical education requirements struggle to meet the developmental needs of students across different levels within the class.	0.189	0.732	0.077
I believe implementing micro-stratified instruction within regular classes is practically feasible.	0.46	0.572	−0.259
I endorse setting differentiated exercise intensity goals for students at different levels.	0.443	0.641	0.247
I believe implementing micro-stratified instruction in regular PE classes will significantly increase teachers' burden of pre-class preparation and classroom management.	0.141	0.115	0.781
I endorse designing differentiated practice content for students at different levels.	0.222	0.555	0.48
I endorse utilizing “individual progress” rather than “uniform standards” to evaluate the learning outcomes of students at different levels.	0.143	0.604	0.321
I believe implementing micro-stratified instruction can effectively enhance classroom participation and enthusiasm among students of various physical fitness levels.	0.36	0.668	0.431
I believe micro-stratified instruction helps improve the physical health status of students with atypically high or low BMI.	0.503	0.5	0.32
I believe Body Mass Index (BMI) is a key criterion for micro-stratified instruction.	0.815	0.187	−0.033
Cardiorespiratory endurance	0.759	0.304	0.164
Muscular strength level	0.801	0.265	0.157
Speed quality level	0.832	0.207	0.159
Flexibility level	0.873	0.063	0.115
Agility level	0.773	0.109	0.341
Sports skills and proficiency	0.609	0.345	0.39
Constructing a micro-stratified framework based on BMI + multidimensional physical fitness data + sports foundation + learning attitude.	0.439	0.461	0.512

Given the high overall Cronbach's α coefficient of 0.957, further exploratory factor analysis and subscale reliability tests were performed to help rule out potential item redundancy that might be masked by such high internal consistency. Consistent with the aforementioned extraction criterion (eigenvalues > 1), the analysis yielded three common factors capturing 66.635% of the cumulative variance. The rotated component matrix suggests the following structure: Factor 1 appears to primarily capture the endorsement of specific physical fitness metrics, such as cardiorespiratory endurance and muscular strength. Factor 2 reflect teaching attitudes aligned with the new curriculum standards and a focus on individual differences. Factor 3 is composed of two items: “significantly increases the burden of pre-class preparation and classroom management” and “constructs a multidimensional comprehensive evaluation framework.” Subscale reliability analyses demonstrated excellent internal consistency for Factor 1 and Factor 2, with Cronbach's α coefficients of 0.925 and 0.891, respectively. The α coefficient for Factor 3 was observed to be 0.580. Within the psychometric literature, Cronbach's α is known to be highly sensitive to the number of items; consequently, two-item subscales typically exhibit structurally lower coefficients. Considering that Factor 3 is primarily intended as a supplementary dimension to explore the practical concerns of frontline educators, and given that the two items share a reasonable logical connection within real-world teaching contexts, this reliability coefficient may be deemed acceptable for the purposes of an exploratory study.

### 2.2 Statistical methods

This study retrospectively extracted physical fitness monitoring data from eighth-grade students at a public middle school in Beijing. An initial dataset of 616 records was collected. During the data cleaning phase, 84 invalid records were excluded. The specific reasons for exclusion were as follows: 31 records were omitted due to absenteeism (e.g., taking leave), resulting in non-participation in the assessments (20 males, 11 females); and 53 records were excluded due to incomplete assessments, which led to missing data for certain test items (23 males, 30 females). The final analytic sample comprised 532 complete and valid records, consisting of 278 males and 254 females. The participants' Body Mass Index (BMI) was categorized strictly in accordance with the *National Student Physical Health Standard (2014 Revision)* issued by the Ministry of Education (16). The grouping criteria were as follows: males were classified into underweight ( ≤ 15.6), normal (15.7–22.5), overweight (22.6–25.2), and obese (≥ 25.3); females were classified into underweight ( ≤ 15.2), normal (15.3–22.2), overweight (22.3–24.8), and obese (≥ 24.9). Data processing and statistical analysis were conducted using SPSS 27.0. Initially, descriptive statistics were performed for the scores of each item, with continuous variables expressed as mean ± standard deviation (Mean ± SD). To examine the differences in the levels of various physical fitness indicators across different BMI groups, a one-way analysis of variance (ANOVA) was performed on the scores of each item, using BMI categorization (underweight, normal, overweight, and obese) as the exploratory factor. Given the substantial disparity in sample sizes across groups and the heterogeneity of variance, the overall significance was determined using Welch's ANOVA to control the risk of Type I error inflation caused by unequal variances (17). Omega-squared (ω^2^) was calculated as the effect size metric, as it yields a less biased estimate than partial Eta-squared under conditions of unequal variances and sample sizes (18). *Post-hoc* multiple comparisons were conducted using the Games-Howell test (19), with the normal-weight group serving as the reference baseline and the significance level set at α = 0.05. To further mitigate potential statistical biases arising from severe sample size disparities across groups, a bootstrap resampling procedure (1,000 resamples) was incorporated. A 95% confidence interval (95% CI) that did not cross zero was adopted as a robust confirmatory criterion for establishing significant inter-group differences, with the two-tailed significance level set at α = 0.05.

Furthermore, to explore the multidimensional structure of the physical fitness parameters and potential latent group typologies, the proposed framework was explicitly designed as a sequential screening and clustering framework. It is crucial to clarify that BMI was utilized exclusively for the preliminary morphological screening categorization and was excluded from the clustering algorithm. The clustering procedure was based exclusively on the six standardized physical fitness performance variables. To align the directionality of the data—ensuring that higher numerical values uniformly indicate superior physical performance across all metrics—timed events where lower scores reflect better performance (i.e., the 50 m, 800 m, and 1000 m runs) were subjected to a reverse transformation (original score × −1) prior to standardization. Subsequently, all six performance variables were transformed using the standard Z-score calculation, *Z* = (*X*−μ)/σ, where *X* represents the raw (or reversed) score, μ represents the sample mean, and σ represents the sample standard deviation. Because standard K-means clustering requires the number of clusters (k) to be specified *a priori*, a TwoStep Cluster analysis was first utilized as an objective heuristic tool. This procedure relies on an automated model-selection algorithm that evaluates alternative clustering solutions based on the Bayesian Information Criterion (BIC) (20) and the ratio of distance measures, identifying the optimal number of clusters for the male and female cohorts as 2 and 3, respectively. Meanwhile, the average Silhouette Measure of Cohesion and Separation are approximately 0.3, indicating modest but acceptable cohesion and separation for an exploratory clustering solution. Subsequently, utilizing these data-driven cluster solutions and the squared Euclidean distance measure, the K-means algorithm was independently executed on the standardized physical fitness variables. The maximum number of convergence iterations was set to 10, ultimately generating an exploratory cluster partition structure to delineate latent trends of micro-heterogeneity in junior high school students' physical health.

The specific test indicators and their corresponding instruments were determined as follows: (1) Body morphology indicators: height (cm) and weight (kg), measured using an electronic stadiometer and scale (Model: HGM-300; Zhengzhou Hengxing Electronic Co., Ltd., China). Body Mass Index (BMI) was calculated as weight (kg) divided by height squared (m^2^). (2) Pulmonary function indicator: vital capacity (ml), measured using a smart electronic spirometer (Model: Taimeiquan Spirometer; Beijing Taimeiquan Technology Co., Ltd., China). To ensure scientific accuracy, students were required to fast and refrain from vigorous exercise for 2 h prior to the test, and to familiarize themselves with the breathing protocol beforehand. During the test, subjects stood upright, took a deep breath, and exhaled as forcefully as possible into a disposable mouthpiece. Each subject performed the test twice with a rest interval of at least 30 s between trials. Each exhalation required a continuous, sudden airflow without any air leakage, and the system automatically recorded the maximum value from the two valid tests (3). Physical fitness indicators: 50-m sprint (seconds), standing long jump (cm), 1,000-m run for males (seconds), 800-m run for females (seconds), pull-ups for males (repetitions), and 1-min sit-ups for females (repetitions). The corresponding testing tools for these indicators were stopwatches, tape measures and jumping mats, standard horizontal bars, and electronic sit-up testers, respectively. Although the sit-and-reach test was conducted using a sit-and-reach tester in compliance with national standards, it was excluded from the subsequent regression and correlation analyses. This decision was made because flexibility lacks a direct physiological and biomechanical correlation with pulmonary function and cardiorespiratory endurance, thereby ensuring the study's core hypotheses remained focused.

Regarding the testing procedures, all assessments were uniformly conducted on the school's standard outdoor synthetic track during morning physical education classes and recess periods. On the day of testing, students wore appropriate athletic clothing and sports shoes, and were instructed to avoid taking the tests on an empty stomach or immediately after a full meal. The testing methods and frequencies for each item were as follows: (1) Height and weight: tested once; students stood barefoot in an upright posture to record the reading. (2) Vital capacity: tested twice, recording the maximum value, with a rest interval of at least 30 s between trials. (3) Sit-and-reach: tested twice, recording the best score, with a 15-s rest between trials. (4) Standing long jump: tested three times, recording the best distance, with a 30-s rest between jumps. (5) 50-meter sprint: tested twice in groups of 2 to 4 from a standing start, timed with a stopwatch. (6) Pull-ups (males): tested once using an overhand grip; a repetition was counted when the chin cleared the bar, and the total completed repetitions were recorded. (7) Sit-ups (females): tested once with a 1-min time limit, recording the number of correctly completed repetitions. (8) 1,000-meter/800-meter run: tested once in groups of 8 to 15 from a standing start, with stopwatch timing recorded to two decimal places in seconds.

Because the study involved adolescent participants, rigorous ethical and data privacy protocols were enforced. Prior to physical fitness testing and secondary data analysis, written informed consent was obtained from the parents or legal guardians of all participating students, and assent was obtained from the students themselves. Strict desensitization and anonymization procedures were implemented: all direct personal identifiers, including names and student ID numbers, were stripped and replaced with randomized study codes prior to data extraction to ensure complete confidentiality. All research procedures fully complied with the ethical guidelines for human subjects as stipulated in the Declaration of Helsinki. Given that the implementing institution of the study had not yet established an independent ethics review committee, the research protocol was specifically commissioned to the authoritative Ethics Review Committee of Beijing Anzhen Hospital, Capital Medical University for formal review and approval [Ethical Approval Number: (2025) Lun Ke Kuai Shen No. (2025-79)].

## 3 Result

### 3.1 Questionnaire survey results

A questionnaire survey was conducted among frontline physical education (PE) teachers to understand their attitudes toward tiered instruction and their current perceptions of physical health evaluation standards. The basic information of the teachers is presented in Table 2. Among the 125 surveyed teachers, there were 76 males (60.8%) and 49 females (39.2%). Regarding professional titles, Second-Grade teachers accounted for the highest proportion with 71 individuals (56.8%), while Senior and Principal Senior teachers combined accounted for only 18.4%. In terms of teaching experience, 83 teachers (66.4%) had 5 years or less of experience, whereas those with over 15 years of experience constituted only 16.8%. Regarding the distribution of teaching locations, 91 teachers (72.8%) were from urban schools, while those in county towns, townships, and rural areas totaled only 34 (27.2%).

**Table 2:** Basic information of the survey respondents.

Item	Options	Number of people
Gender	Male	76 (60.8%)
	Female	49 (39.2%)
Professional title	Principal senior teacher	6 (4.8%)
	Senior teacher	17 (13.6%)
	First-grade teacher	16 (12.8%)
	Second-grade teacher	71 (56.8%)
	Third-grade teacher	15 (12%)
Teaching experience	≤ 5 years	83 (66.4%)
	6–10 years	14 (11.2%)
	11–15 years	7 (5.6%)
	>15 years	21 (16.8%)
Teaching location	Urban	91 (72.8%)
	County town	22 (17.6%)
	Township/Rural	12 (9.6%)

The survey results regarding teachers' attitudes toward tiered instruction are shown in Table 3. On the five-point Likert scale, 1–5 represent responses ranging from “strongly disagree” to “strongly agree.” The data indicate that teachers have a high overall acceptance of the necessity of tiered instruction, with a total average score of 4.41 and the mean of each item exceeding 4.0. This result suggests that most teachers are not only well aware of the curriculum standards' requirements for addressing individual differences, but they have also profoundly perceived the heterogeneity of students within their classes in practice, leaning toward comprehensive evaluation in their assessment concepts. Furthermore, the mean scores for items 4 and 6 were 4.02 and 4.32, respectively. Although these means remain at a relatively high level, they are lower compared to other items, and the combined proportion of teachers selecting “neutral” and “disagree” reached 23.2%. This indicates that despite acknowledging the conceptual value of tiered instruction, teachers still harbor certain concerns regarding its operability and feasibility under current realistic conditions.

**Table 3:** Survey on teachers' attitudes toward the implementation of tiered instruction.

**Item no**.	**Options**	1(N)	2(N)	3(N)	4(N)	5(N)	Mean score
1	I understand the basic curriculum concept of “focusing on students' individual differences and ensuring every student benefits” in the new curriculum standards.	0	1	9	32	83	4.58
2	I believe there are differences in the physical fitness and athletic foundations of students in the same class, which require differentiated treatment.	1	2	6	34	82	4.55
3	I believe uniform physical education teaching requirements are difficult to meet the development needs of students at different levels in the whole class.	3	2	11	31	78	4.43
4	I believe implementing tiered instruction in regular classes is practically feasible.	5	6	18	48	48	4.02
5	I agree with setting differentiated exercise intensity targets for students at different levels.	1	4	10	39	71	4.4
6	I believe implementing tiered instruction in regular physical education classrooms will significantly increase teachers' pre-class preparation and classroom management burdens.	0	2	15	49	59	4.32
7	I agree with designing differentiated practice content for students at different levels.	0	2	8	49	66	4.43
8	I agree with using the “extent of individual progress” rather than “uniform standards” in evaluation to measure the learning effectiveness of students at different levels.	0	1	9	37	78	4.54
9	I believe implementing tiered instruction can effectively enhance the classroom engagement and enthusiasm of students with different physical fitness levels.	1	0	10	41	73	4.48
10	I believe tiered instruction helps improve the physical health status of students with high or low BMI.	1	1	18	42	63	4.32

The questionnaire uses a Likert 5-point scale, where 1–5 represent from “strongly disagree” to “strongly agree”, respectively. The numbers in parentheses indicate the number of people who selected that option.

As shown in Table 4, the total average score for teachers' acceptance of student stratification indicators was 4.07, indicating a high overall level of agreement, though significant divergence existed across dimensions. The most highly endorsed item was “evaluation criteria should be constructed by integrating BMI, multidimensional physical fitness data, athletic foundation, and learning attitude,” which achieved an average score of 4.50, with 62.4% of respondents giving it the highest level of agreement (“strongly agree”). This was followed in priority by “mastered motor skills and proficiency” (4.28). Among traditional physical fitness indicators, speed, cardiorespiratory endurance, and strength received relatively higher acceptance, whereas flexibility (3.82) and BMI as a single item (3.89) had the lowest acceptance. These results indicate that most frontline PE teachers prefer establishing a multidimensional comprehensive evaluation system comprising “physical fitness foundation + motor skills + learning attitude.” However, to explore the weight distribution of this conceptual preference in realistic decision-making, the study further introduced an indicator importance ranking question, requiring respondents to select and rank the top three most important indicators. The ranking results showed that the composite score for cardiorespiratory endurance was 6.06, ranking first; the composite score for BMI was 54, ranking second; and the composite score for specific sports skills was 3.98, ranking third. Notably, although the composite score for BMI ranked second, 65.85% of teachers ranked BMI in the first place, whereas only 29.47% of teachers selected cardiorespiratory endurance as their first choice, with 49.47% placing it second (Table 5). Based on the comprehensive questionnaire survey results, it can be concluded that although the teacher cohort widely recognizes the objective existence of students' physical heterogeneity and the necessity of differentiated interventions, obvious practical concerns remain when specifically implementing tiered instruction. Regarding the selection of stratification indicators, teachers generally lean toward choosing multidimensional stratification indicators, and the majority of teachers opt to position BMI as the most important stratification indicator.

**Table 4:** Survey on acceptance of stratification indicators.

Options	1(N)	2(N)	3(N)	4(N)	5(N)	Mean score
Body mass index (BMI)	2	11	25	48	39	3.89
Cardiorespiratory endurance level	1	2	28	55	39	4.03
Muscular strength level	1	3	31	50	40	4
Speed quality level	1	1	29	51	43	4.07
Flexibility level	2	8	38	39	38	3.82
Agility level	2	7	31	43	42	3.93
Mastered motor skills and proficiency	1	1	16	51	56	4.28
Constructing a stratification framework by integrating BMI + multidimensional physical fitness data + athletic foundation + learning attitude.	1	0	12	34	78	4.5

The questionnaire uses a Likert 5-point scale, where 1 to 5 represent from “strongly disagree” to “strongly agree”, respectively. The numbers in parentheses indicate the number of people who selected that option.

**Table 5:** Ranking of stratification indicator importance.

Options	Composite score	1st place	2nd place	3rd place
Cardiorespiratory endurance	6.06	28 (29.47%)	47 (49.47%)	16 (16.84%)
Body composition (BMI)	5.28	54 (65.85%)	11 (13.41%)	9 (10.98%)
Specific sports skills	3.98	23 (30.26%)	11 (14.47%)	14 (18.42%)
Strength quality	3.72	8 (12.7%)	20 (31.75%)	31 (49.21%)
Speed quality	3.3	4 (6.45%)	10 (16.13%)	23 (37.1%)
Agility quality	2.12	0 (0%)	10 (20.41%)	4 (8.16%)
Sports self-confidence	2.02	4 (8.33%)	9 (18.75%)	14 (29.17%)
Flexibility quality	1.69	2 (5%)	0 (0%)	6 (15%)
Technical and tactical application	1.35	2 (5.26%)	6 (15.79%)	6 (15.79%)

BMI, body mass index. The composite score is calculated based on the frequencies of each indicator being selected in the 1st, 2nd, and 3rd places and assigned corresponding weights; a higher score indicates a higher importance ranking for the indicator.

### 3.2 Descriptive statistical results of physical fitness monitoring data

The subjects involved in the data sample were all eighth-grade students, aged approximately 14 years. Their basic information is shown in Table 6. The descriptive analysis results indicate that the mean vital capacity of the obese male group (3,300.77 ± 584.06 ml) was the highest among the four groups, but their performance across multiple other physical fitness indicators was the worst. In contrast, the normal-weight male group performed the best in the 50-m dash (8.05 ± 0.85 s), standing long jump (199.30 ± 24.29 cm), and pull-ups (5.98 ± 7.36 reps). The indicators of the overweight male group fell between those of the normal and obese groups, while the underweight group was slightly inferior to the normal group in vital capacity (2,547.29 ± 413.60 ml) and the 1,000-m run (256.67 ± 27.14 s).

**Table 6:** Basic information of male and female students.

Gender	Height (cm)	Weight (kg)	BMI (kg/m^2^)
Male	163.297 ± 8.052	54.759 ± 12.884	20.373 ± 3.720
Female	160.381 ± 5.910	49.79 ± 10.508	19.261 ± 3.427

BMI, body mass index. *N* represents the sample size of each group. All continuous physical fitness indicator data are expressed as Mean ± Standard Deviation (Mean ± SD).

The performance of the female cohort differed somewhat from that of the males. The obese female group performed the worst among the four groups in the 50-m dash (9.94 ± 0.66 s), standing long jump (149.88 ± 17.06 cm), 800-m run (265.18 ± 32.91 s), and sit-ups (39.12 ± 9.87 reps), but their vital capacity (2,802.29 ± 383.60 ml) was not the lowest, being only slightly lower than that of the overweight group (2,843.44 ± 464.62 ml). The normal-weight female group performed the best in the 800-m run (220.92 ± 21.44 s), sit-and-reach (20.19 ± 5.78 cm), and sit-ups (48.42 ± 8.82 reps), but was slightly inferior to the underweight group in vital capacity (2,572.66 ± 410.12 ml) and the 50-m dash (8.93 ± 0.61 s).

### 3.3 One-way analysis of variance

To explore the differences in physical fitness across BMI categories, a one-way analysis of variance was conducted. Complete descriptive statistics, including specific group sizes (*n*), are provided in Table 7. Due to the heterogeneity of variance confirmed by Levene's test, overall significance was evaluated using Welch's ANOVA. Exact test statistics, degrees of freedom (*df*1, *df*2), exact *p*-values, and effect sizes (ω^2^) for all metrics are fully detailed in Table 8. The analysis revealed significant inter-group differences across all physical fitness test items for both the male and female cohorts (*p* < 0.05). The effect sizes (ω^2^) ranged from 0.005 to 0.179, indicating small to large effects depending on the specific fitness metric. The most substantial physiological impacts were observed in the 800-m run (ω^2^ = 0.179) and 50-meter dash (ω^2^ = 0.145) for the female cohort, and vital capacity (ω^2^ = 0.085) for the male cohort. Using the normal-weight group as the reference baseline, *post-hoc* multiple comparisons were conducted utilizing the Games-Howell test, corroborated by Bootstrap 95% confidence intervals for the mean differences. In the male cohort, the normal-weight group significantly outperformed the underweight, overweight, and obese groups in vital capacity, the 50-meter dash, and the standing long jump. Furthermore, physical performance was significantly inferior to the normal-weight baseline for the obese group in the 1,000-m run (*p* = 0.026), and for both the overweight (*p* = 0.005) and obese groups (*p* < 0.001) in pull-ups (see Table 9). In the female cohort, the underweight group exhibited significant differences in vital capacity (*p* < 0.001) and the sit-and-reach (95% CI = 0.017, 7.056) compared to the normal-weight baseline. The overweight group demonstrated significant deviations in vital capacity (*p* = 0.034) and the standing long jump (*p* = 0.040). Notably, the obese female group experienced a comprehensive and significant decline across multiple core qualities, including the 50-m dash (*p* < 0.001), standing long jump (*p* < 0.001), 800-m run (*p* < 0.001), and sit-ups (*p* = 0.007; see Table 10).Ultimately, these findings confirm that physical fitness scores exhibit distinct gender and morphological variations, with the obese cohort demonstrating the most pronounced performance deficits.

**Table 7:** Descriptive statistics of male and female students' physical fitness test scores.

Gender	BMI category	N	Vital capacity (ml)	50-m dash (s)	Standing long jump (cm)	Sit-and-reach (cm)	1,000/800-m run (s)	Sit-ups/pull-ups (reps)
Male	Underweight	21	2,547.29 ± 413.60	8.54 ± 0.84	189.00 ± 17.90	8.64 ± 7.09	256.67 ± 27.14	5.19 ± 6.99
Male	Normal	185	2,984.03 ± 542.30	8.05 ± 0.85	199.30 ± 24.29	10.77 ± 8.09	258.84 ± 36.44	5.98 ± 7.36
Male	Overweight	42	3,185.45 ± 552.00	8.39 ± 0.85	191.81 ± 22.71	12.44 ± 4.87	266.33 ± 34.44	2.93 ± 4.56
Male	Obese	30	3,300.77 ± 584.06	8.78 ± 1.69	184.70 ± 28.74	9.38 ± 8.91	288.37 ± 52.36	1.37 ± 3.91
Female	Underweight	19	2,177.84 ± 336.56	8.86 ± 0.50	175.53 ± 13.82	16.65 ± 7.77	219.05 ± 20.87	49.95 ± 8.57
Female	Normal	191	2,572.66 ± 410.12	8.93 ± 0.61	173.62 ± 17.12	20.19 ± 5.78	220.92 ± 21.44	48.42 ± 8.82
Female	Overweight	27	2,843.44 ± 464.62	9.14 ± 0.61	163.37 ± 17.93	17.94 ± 5.89	232.00 ± 32.30	46.59 ± 7.69
Female	Obese	17	2,802.29 ± 383.60	9.94 ± 0.66	149.88 ± 17.06	16.05 ± 7.41	265.18 ± 32.91	39.12 ± 9.87

BMI, body mass index. *N* represents the sample size of each group. All continuous physical fitness indicator data are expressed as Mean ± Standard Deviation (Mean ± SD).

**Table 8:** Welch's ANOVA results of physical fitness test scores among male and female students across BMI categories.

Gender	Test item	df1	df2	*F*	ω^2^	*p*-value
Male	Vital capacity	3	58.495	12.328	0.085	< 0.001
	50 m sprint	3	54.447	4.618	0.054	0.006
	Standing long jump	3	58.761	4.114	0.035	0.01
	Sit-and-reach	3	59.105	2.321	0.005	0.084
	1,000 m run	3	57.952	3.379	0.047	0.024
	Pull-ups	3	63.155	9.769	0.048	< 0.001
Female	Vital capacity	3	38.318	13.332	0.11	< 0.001
	50 m sprint	3	38.062	12.919	0.145	< 0.001
	Standing long jump	3	38.339	12.044	0.12	< 0.001
	Sit-and-reach	3	36.057	3.466	0.041	0.026
	800 m run	3	35.797	10.427	0.179	< 0.001
	Sit-ups	3	37.731	5.134	0.06	0.004

Due to the substantial disparity in sample sizes across BMI groups and the failure to meet the assumption of homogeneity of variance (*p* < 0.05), Welch's ANOVA was employed. df1 represents the between-group degrees of freedom, and df2 represents the adjusted residual degrees of freedom. The *F*-value is the Welch statistic, and ω^2^ represents the fixed-effect size.

**Table 9:** Results of *Post-Hoc* multiple comparisons for male students.

Indicator	Comparison group	*p*-value	95% CI
Vital capacity	Underweight	< 0.001	(230.707, 627.476)
	Overweight	0.152	(−389.092, −23.441)
	Obese	0.04	(−541.128, −110.064)
50-m dash	Underweight	0.082	(−0.9173, −0.1417)
	Overweight	0.112	(−0.6136, −0.047)
	Obese	0.117	(−1.3666, −0.168)
Standing long jump	Underweight	0.1	(2.264, 18.895)
	Overweight	0.237	(0.178, 15.439)
	Obese	0.057	(4.399, 24.781)
Sit-and-reach	Underweight	0.581	(−1.2006, 5.2616)
	Overweight	0.308	(−3.5329, 0.1981)
	Obese	0.852	(−2.255, 4.5056)
1,000-m run	Underweight	0.987	(−11.339, 14.089)
	Overweight	0.592	(−19.333, 3.546)
	Obese	0.026	(−49.676, −11.314)
Pull-ups	Underweight	0.961	(−2.816, 3.677)
	Overweight	0.005	(1.325, 4.785)
	Obese	< 0.001	(2.853, 6.228)

This table presents the results of *post-hoc* multiple comparisons using the Games-Howell test. All comparisons use the “normal weight group” as the reference baseline. The 95% CI is the confidence interval obtained after 1,000 resamples using the Bootstrap method.

**Table 10:** Results of *Post-Hoc* multiple comparisons for female students.

Indicator	Comparison group	*p*-value	95% CI
Vital capacity	Underweight	< 0.001	(241.684, 557.495)
	Overweight	0.034	(−449.305, −93.236)
	Obese	0.121	(−415.229, −30.539)
50-m dash	Underweight	0.947	(−0.30032, 0.19)
	Overweight	0.334	(−0.00686, 0.46207)
	Obese	< 0.001	(0.68092, 1.36353)
Standing long jump	Underweight	0.943	(−8.489, 4.436)
	Overweight	0.04	(2.795, 17.541)
	Obese	< 0.001	(14.899, 31.618)
Sit-and-reach	Underweight	0.246	(0.0173, 7.0558)
	Overweight	0.263	(−0.2248, 4.8637)
	Obese	0.15	(0.134, 7.8536)
800-m run	Underweight	0.982	(−11.925, 8.373)
	Overweight	0.327	(−0.094, 24.194)
	Obese	< 0.001	(29.378, 60.337)
Sit-ups	Underweight	0.88	(−5.878, 2.557)
	Overweight	0.672	(−1.141, 4.934)
	Obese	0.007	(4.624, 14.341)

This table presents the results of *post-hoc* multiple comparisons using the Games-Howell test. All comparisons use the “normal weight group” as the reference baseline. The 95% CI is the confidence interval estimated using the Bootstrap method (1,000 iterative resamples).

### 3.4 Cluster analysis

To further explore the potential stratification characteristics of male and female students' physical health scores, the study first performed *Z*-score standardization on all physical fitness test scores, and subsequently included them as continuous variables in a TwoStep Cluster analysis. Based on the Bayesian Information Criterion (BIC) assessment, the optimal number of clusters for males was determined to be 2, and the optimal number of clusters for the female cohort was determined to be 3. The subsequent K-means cluster analysis, based on the aforementioned results, executed iterative clustering of *K* = 2 and *K* = 3 for the male and female samples, respectively. To further validate the structural stability and group separation of the *K*-means solution—and to address the feature overlap reflected by the average silhouette coefficient—a Discriminant Analysis was conducted. Utilizing the cluster labels generated by the analysis as the grouping variable and the standardized physical fitness indicators as predictors, the functions at group centroids revealed significant spatial separation. Specifically, the centroid coordinates for the two male clusters on the primary discriminant function were 0.967 and −1.804, indicating a substantial inter-class distance. The three female clusters also exhibited a vast spatial centroid span across their respective discriminant functions (Tables 11, 12). Furthermore, leave-one-out cross-validation results indicated that the overall correct classification rates for the male and female solutions reached 96.0 and 95.7%, respectively. These high classification accuracies, combined with the significant group centroid distances, collectively demonstrate that the extracted physical fitness typologies possess acceptable internal validity and within-sample classification stability. However, given the modest silhouette score (~0.30), these findings represent an exploratory typology rather than a universally generalizable classification. Combining the BMI cross-distribution of each clustered population (Figure 1) and the Z-score radar chart of physical fitness indicators (Figure 2), the male cohort presented two major clusters with distinct characteristics. For Cluster 1 (*n* = 182), as can be seen from Figure 1, there were 132 normal-weight students in this cluster, accounting for approximately 72.5%. The radar chart in Figure 2 intuitively shows that the Z-scores of this group of males in all physical fitness test items, such as vital capacity, the 50-m dash, 1,000-meter run, standing long jump, sit-and-reach, and pull-ups, were all greater than 0. This indicates that the students in this cluster not only have proportionate body types, but are also above the overall mean in terms of speed, endurance, lower limb explosive power, and upper limb strength, demonstrating a comprehensive and balanced physical fitness advantage. For Cluster 2 (*n* = 96), the *Z*-scores of this group in core sports items such as the standing long jump, pull-ups, 50-m dash, and 1,000-m run all presented obvious negative values. This data characteristic reveals a comprehensive lag in this cohort regarding overcoming their own gravity as well as in absolute speed and endurance performance. The clustering characteristics of the female cohort were manifested as follows: Cluster 1 consisted of 102 people, among which 87 were normal-weight females, accounting for over 85.3%, but there was only 1 obese female. The radar chart showed that, except for vital capacity which was roughly at the overall average level, the Z-scores of this group of females in the 800-m run, 50-m dash, standing long jump, sit-ups, and sit-and-reach were all positive values, demonstrating a comprehensive and balanced physical fitness advantage. Cluster 2 had 28 people, including 11 each in the obese group and the normal weight group, and 6 in the overweight group. The scores of this group, except for vital capacity, were all below the mean, and their Z-scores for the 800-m run, 50-m dash, standing long jump, and sit-ups were all smaller than the other two clusters, even falling sharply below −1, demonstrating the cohort characteristic of “vital capacity advantage—physical fitness weakness.” Cluster 3 consisted of 124 people, of which 93 were in the normal group (accounting for 75%), 12 were in the overweight group, and only 5 were in the obese group; its characteristic was that all scores were slightly below the mean, presenting a characteristic of being “comprehensively weak.”

**Table 11:** Functions at group centroids for male and female clustering groups.

Gender	Cluster group	Function 1	Function 2
Male	Cluster 1	0.967	—
	Cluster 2	−1.804	—
Female	Cluster 1	2.05	0.302
	Cluster 2	−3.971	0.982
	Cluster 3	−0.789	−0.47

**Table 12:** Standardized means (*Z*-scores) of fitness components at final cluster centers.

Fitness component (*Z*-score)	Male cluster 1	Male cluster 2	Female cluster 1	Female cluster 2	Female cluster 3
Vital capacity	0.24	−0.454	0.334	0.543	−0.397
50 m sprint (reversed)^*^	0.495	−0.938	0.707	−1.602	−0.22
Standing long jump	0.485	−0.92	0.8	−1.663	−0.282
Sit-and-reach	0.261	−0.494	0.565	−0.61	−0.327
Pull-ups/sit-ups	0.309	−0.586	0.42	−1.3	−0.052
1,000 m/800 m Run (Reversed)^*^	0.501	−0.949	0.476	−1.65	−0.019

Timed running events were reverse-standardized so that higher positive values consistently indicate superior athletic performance.

**Figure 1:**
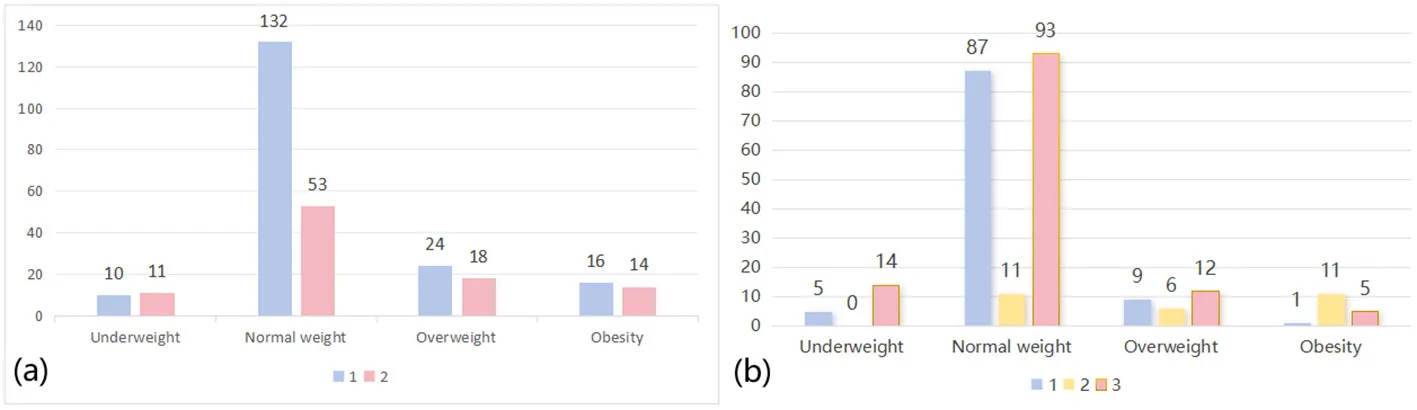
Sample distribution of male and female *K*-means clustering groups under four BMI categories. The figure illustrates the sample distribution quantities of males (2 clusters in total) and females (3 clusters in total) under four BMI categories (underweight, normal, overweight, and obese) following K-means clustering based on multidimensional physical fitness data. **(a)** is for males, and **(b)** is for females.

**Figure 2:**
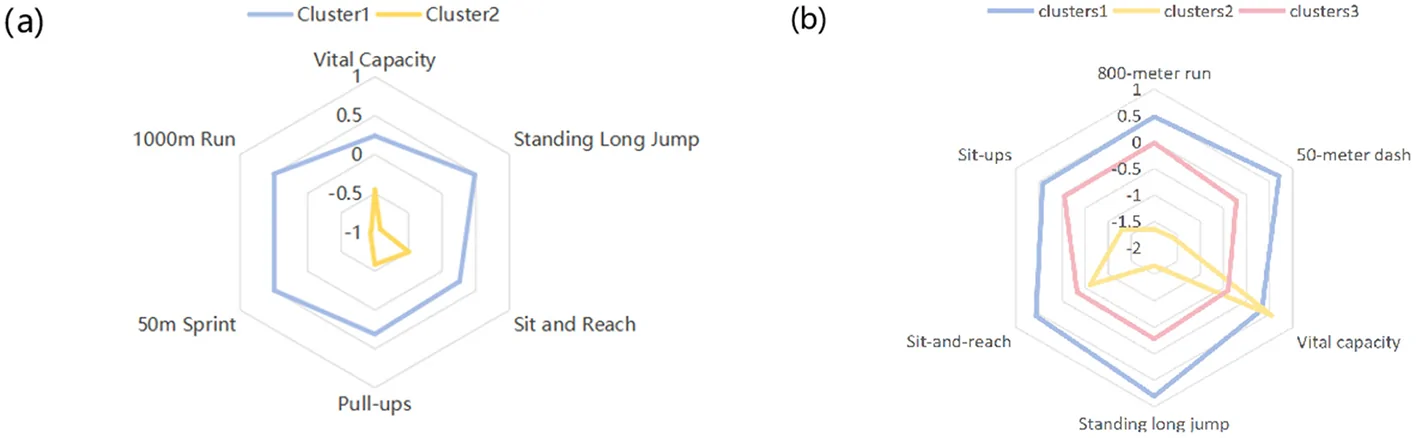
Radar chart of mean *Z*-scores on six physical fitness test items for male and female clustering groups. Male Cluster 1 (*n* = 182, 65.5%), Male Cluster 2 (*n* = 96, 34.5%); Female Cluster 1 (*n* = 102, 40.2%), Female Cluster 2 (*n* = 28, 11.0%), Female Cluster 3 (*n* = 124, 48.8%). The radar chart displays the mean standardized scores (*Z*-scores) of each clustering group across various physical fitness test items. A *Z*-score = 0 represents the overall average level; a *Z*-score > 0 represents performance superior to the mean, and a *Z*-score < 0 represents performance inferior to the mean. The 50-m dash and 1,000/800-m run have been reverse-standardized, meaning the further outward the value, the better the performance. **(a)** is for males, and **(b)** is for females. To maintain visual legibility across overlapping multidimensional polygons, error bars/confidence intervals are omitted from this chart; exact standardized centroids for each physical fitness component are detailed in Table 12.

## 4 Discussion

Against the backdrop of declining adolescent physical health, this study addresses the gap between the theory and practice of differentiated instruction in junior high school PE classrooms. To resolve this practical dilemma, we constructed a composite stratification framework integrating BMI preliminary screening with multidimensional physical fitness data clustering. This framework adapts Tomlinson's theory of differentiated instruction for localized large-class environments, providing an exploratory framework to map baseline physical health interventions rather than a clinically validated strategy.

The questionnaire survey results indicate that frontline teachers exhibit a high degree of acceptance for the concept of tiered instruction (mean score 4.55). This demonstrates that with the in-depth implementation of the Physical Education and Health Curriculum Standards for Compulsory Education, the developmental evaluation concept of “focusing on individual differences and promoting students' comprehensive development” has formed a broad consensus among the teacher cohort. Most teachers have acutely observed that within regular administrative classes, there is enormous heterogeneity in students' body morphology, cardiorespiratory endurance, muscular strength, and motor skill foundations, and the traditional “one-size-fits-all” teaching approach is no longer able to meet the health demands of students at different levels.

However, teachers' acceptance of the practical feasibility of implementing tiered instruction in the regular large-class teaching process showed a decline (mean score 4.02). This indicates that teachers are not denying the value of tiered instruction, but rather reflecting the dilemma of large-class teaching in the current basic education stage. As pointed out by other studies, time constraints, spatial crowding, and management load are common barriers for global PE teachers in implementing individualized instruction (21). Because practical dilemmas such as large class sizes and limited facilities cannot undergo fundamental changes in the short term, the starting point of this study is precisely to explore a stratification framework that accommodates both group commonalities and individual differences, without substantially increasing teachers' lesson preparation and management loads, in the hope of finding an optimal solution amid the current teaching dilemmas.

When exploring the specific selection of stratification indicators, the questionnaire data showed that 62.4% of teachers recognized the value of multidimensional comprehensive evaluation. However, in the ranking of indicator importance, 65.85% of teachers still regarded Body Mass Index (BMI) as their primary basis for intervention. It is crucial to clarify that this preference should *not* be interpreted as evidence that BMI is the most scientifically appropriate or comprehensive primary criterion. Rather, this choice is heavily driven by practical feasibility, operational constraints, and institutional norms embedded within current national surveillance systems. In large-class settings, BMI serves as a zero-cost, highly reliable metric for rapid triage. While the subsequent one-way analysis of variance (ANOVA) confirmed significant inter-group differences across BMI categories—validating its value in the preliminary screening stage—it also glaringly exposed the limitations of BMI as a solitary metric. Specifically, BMI cannot differentiate fat-free mass from adiposity, and it is heavily influenced by biological maturation in this age group. Taking the male cohort as an example, the absolute vital capacity of males in the obese group ranked first among all groups, but their performance in events requiring the overcoming of one's own gravity (such as the 50-m dash, standing long jump, and pull-ups) was significantly inferior to that of the normal-weight group (*p* < 0.05). This phenomenon corroborates the “inverted U-shaped relationship” between adolescents' BMI and the Physical Fitness Index (PFI). Because BMI fails to distinguish between fat mass (dead weight) and fat-free mass, and ignores individual maturational stages, a single morphological classification easily masks the micro-heterogeneity of students' specific athletic qualities. Therefore, our proposed sequential framework explicitly restricts BMI to a preliminary screening role, utilizing multidimensional data clustering to correct morphological misjudgments and reveal true physical profiles.

The results of the one-way analysis of variance (ANOVA) indicated that there were significant inter-group differences in various physical fitness scores among different BMI groups for both males and females, especially between the obese group and the normal weight group. This confirms the foundational screening value of BMI in the preliminary screening stage, echoing the phenomenon that most teachers acknowledge BMI as the most important stratification indicator. However, these physical fitness differences expose a highly complex imbalance in specific athletic phenotypes. For example, while obese males exhibited the highest absolute vital capacity among all groups, their performance in the 50-m dash, standing long jump, pull-ups, and 1,000-m run was significantly inferior to that of the normal-weight group (*p* < 0.05). This corroborates the established “inverted U-shaped relationship” between adolescent BMI and the Physical Fitness Index (PFI) (22). From a physiological and biomechanical perspective, obese adolescents often exhibit higher absolute vital capacity due to expanded thoracic volume, alongside greater absolute lower-limb strength developed to support their body mass during daily activities. However, an extremely high BMI indicates that fat mass accumulation has vastly outpaced fat-free mass growth. During relative strength and explosive power events requiring individuals to overcome their own gravity (e.g., the 50-m dash or pull-ups), excess adipose tissue acts as dead weight. It provides no contractile force and instead creates a massive biomechanical burden, sharply reducing athletic performance (23). Modern public health interventions emphasize that a high level of physical fitness (Fitness) can largely offset the metabolic risks brought about by high BMI (Fatness).

However, physical fitness differentiation exists across different weight groups. For instance, in the normal weight female cohort, the proportion of individuals with all physical fitness scores below the mean reaches 48%, while those comprehensively outperforming the mean account for 46%. The male cohort presents a similar pattern; although 71.4% of the normal weight group comprehensively outperforms the mean, 28.6% of the students still exhibit varying degrees of shortcomings in their physical fitness test scores. This result indicates that a single morphological classification easily masks the micro-heterogeneity of students in specific athletic qualities (24). Modern public health interventions emphasize that a high level of physical fitness (Fitness) can largely offset the metabolic risks of high BMI (Fatness) (25). Therefore, just as most teachers expected in the questionnaire, physical education must abandon its overreliance on a single indicator and shift toward a “BMI + multidimensional motor skill performance” composite stratification framework.

This study utilized *Z*-score standardization and employed the *K*-means clustering algorithm to precisely extract highly condensed physical fitness clusters, thereby constructing the “BMI preliminary screening + multidimensional physical fitness data” composite stratification framework. The results revealed the patterns of physical stratification and gender heterogeneity. The male cohort presented a polarized trend, namely the “balanced physical fitness advantage group” and the “weak strength and large body weight characteristic group.” The female cohort was subdivided into three more complex categories: “balanced advantage,” “comprehensive weakness,” and “vital capacity advantage—physical fitness weakness.” While ensuring the scientific validity and precision of the stratification, this stratification framework strictly limits the “teaching tiers” that teachers need to manage in large classes to 2 or 3, maximally controlling the management load and serving as the key to breaking the “knowing-doing gap” among frontline physical education teachers.

In practical teaching scenarios, this stratification framework provides data support for constructing an efficient, clear, and highly operable implementation pathway. First, BMI is used to quickly screen out students with extreme body types, supplemented by targeted dietary and behavioral interventions outside of class. Second, differentiated exercise prescriptions directly corresponding to different clusters are implemented in regular classes. Because students in “Cluster 1” (both males and females) already possess a good cardiorespiratory and muscular foundation, higher-order motor skills and high-intensity interval training (HIIT) content can be moderately introduced during teaching while maintaining regular exercise intensity. Studies have proven that through alternating intense exercise and brief rest in a short period, HIIT can significantly enhance adolescents' maximal oxygen uptake (VO2 max) and generate more prolonged excess post-exercise oxygen consumption (EPOC), thereby maximizing the stimulation of their physical and metabolic potential within limited class time (26, 27). For students in “Cluster 2” (both males and females), it is recommended to prudently conduct (or minimize) high-impact running and jumping events to protect their joints, instead focusing on low-impact aerobic exercises and foundational strength awakening of core muscle groups (28). More importantly, providing this cohort with sports events they are competent in is a crucial psychological intervention to prevent “learned helplessness” and rebuild their athletic confidence and intrinsic motivation (29). For the third category of the female cohort, classroom interventions should appropriately reduce the proportion of basic aerobic endurance running while focusing on the specific activation of core muscle groups and lower limb explosive power to compensate for their “lack of strength” shortcoming. As pointed out by the international consensus on adolescent resistance training, a single aerobic endurance intervention is insufficient to comprehensively improve adolescents' muscular imbalances; it is necessary to integrate targeted neuromuscular awakening and core resistance training to effectively address their relative strength deficiencies and reduce the risk of sports injuries (30). Furthermore, while teachers might eventually rely on staged retest data over the semester to track students' dynamic migrations across clusters, it is imperative to acknowledge the boundaries of this cross-sectional observational design. Because this research relies exclusively on cross-sectional observational data, it does not involve prospective interventions, safety monitoring, or longitudinal tracking of pedagogical outcomes. Consequently, the tailored exercise prescriptions proposed herein serve strictly as an exploratory framework derived from data profiling, rather than a clinically validated educational strategy. Moving forward, any transition from this theoretical baseline to a truly actionable instructional pathway will require rigorous empirical validation through randomized controlled trials.

## 5 Limitations

The survey sample predominantly comprised younger, urban educators, with a notable underrepresentation of rural and senior teaching staff, which may introduce selection bias. The cross-sectional nature of the data inherently limits the capacity to infer long-term causal relationships. Third, the proposed instructional stratification framework currently serves as a theoretically derived framework grounded in existing literature. Its practical pedagogical efficacy, the longitudinal developmental stability of students within these typologies, and its broader generalizability across diverse regions and school contexts remain to be corroborated by prospective randomized controlled trials (RCTs) and external validations. While discriminant analysis confirmed within-sample classification accuracy, the modest silhouette score (~0.30) necessitates caution regarding the generalizability of these clusters. Future research must employ rigorous external stability testing, such as bootstrap resampling or cross-cohort validation, to confirm the structural stability of these typologies beyond the current sample. Future investigations should endeavor to broaden the sampling scope to enhance external validity. Furthermore, conducting longitudinal RCTs within authentic educational settings is highly recommended to empirically assess the framework's potential impact on adolescents' physical fitness and metabolic parameters, thereby generating a more robust evidence base. Finally, owing to objective constraints, the physical fitness data were extracted exclusively from a specific grade cohort within a single middle school in Beijing. This localized sampling may harbor underlying socioeconomic or geographical biases, which tentatively restricts the direct extrapolation of the current clustering outcomes to a national scale or varying institutional typologies.

## 6 Conclusion

The composite stratification framework—integrating initial BMI screening with multidimensional physical fitness data clustering—functions as an exploratory framework for identifying heterogeneous fitness profiles in large-scale physical education classes. By breaking the path dependence on a single morphological indicator, this approach effectively captures the micro-level heterogeneity of adolescent physical capabilities. However, relying solely on cross-sectional observational data, this study lacks prospective interventions and safety monitoring. Rigorous longitudinal validation is required before this exploratory framework can be adopted as a validated educational strategy.

## Data Availability

The original contributions presented in the study are included in the article/Supplementary material, further inquiries can be directed to the corresponding author.
