# Supplementary material for: A data-driven stratification framework for physical fitness in junior high school students: a composite approach based on BMI and multidimensional data
Source: Front Public Health. 2026 Sep 28;14:1959431. doi: 10.3389/fpubh.2026.1959431 (PMC13654965; doi:10.3389/fpubh.2026.1959431)
Supplement: Supplementary file 1 [file Data_Sheet_1.pdf]

Expert Questionnaire on the Application of Classroom Micro-Tiered Instruction in Precisely Promoting Adolescent Physical Health

Dear Physical Education Teachers: Hello! The purpose of this questionnaire is to understand your views and practical intentions regarding the implementation of "micro-tiered instruction" in physical education (PE) classrooms, providing research references for promoting the precise improvement of adolescent physical health. Every choice you make will provide a critical basis for the transformation of adolescent PE classrooms in our country. This questionnaire is strictly for academic research and is filled out anonymously. We sincerely thank you for your strong support and intellectual contribution!

Note: "Classroom micro-tiered instruction" in this questionnaire refers to implicitly dividing the students of a class into 2–4 learning groups based on their current physical fitness, skills, or body composition, without breaking up the administrative class. It involves implementing differentiated instruction in teaching objectives, practice content, exercise load, and evaluation methods, and dynamically adjusting the groups based on student progress.

1. Gender [Single choice] *

○ Male

○ Female

2. Your professional title is [Single choice] *

○ Principal Senior Teacher

○ Senior Teacher

○ First-Grade Teacher

○ Second-Grade Teacher

○ Third-Grade Teacher

3. Your teaching experience is [Single choice] *

○ ≤ 5 years

○ 6~10 years

○ 11~15 years

○ > 15 years

4. The location where you teach is [Single choice] *

○ Urban

○ County Town

○ Township/Rural

5. Based on your professional knowledge, please indicate your level of agreement with the following statements. (The following are all single-choice questions. Please select the option that best matches your true feelings by checking "√" from "1=Strongly disagree, 2=Disagree, 3=Neutral/Unsure, 4=Agree, 5=Strongly agree".) [Matrix scale question] *

	1	2	3	4	5
I understand the basic curriculum concept of "focusing on students' individual differences and ensuring every student benefits" in the new curriculum standards.	○	○	○	○	○
I believe there are differences in the physical fitness and athletic foundations of students in the same class, which require differentiated treatment.	○	○	○	○	○
I believe uniform physical education teaching requirements are difficult to meet the development needs of students at different levels in the whole class.	○	○	○	○	○
I believe implementing micro-tiered instruction in regular classes is practically feasible.	○	○	○	○	○
I agree with setting differentiated exercise intensity targets for students at different levels.	○	○	○	○	○

	1	2	3	4	5
I believe implementing micro-tiered instruction in regular PE classrooms will significantly increase teachers' pre-class preparation and classroom management burdens.	○	○	○	○	○
I agree with designing differentiated practice content for students at different levels.	○	○	○	○	○
I agree with using the "extent of individual progress" rather than "uniform standards" in evaluation to measure the learning effectiveness of students at different levels.	○	○	○	○	○
I believe implementing micro-tiered instruction can effectively enhance the classroom engagement and enthusiasm of students with different physical fitness levels.	○	○	○	○	○
I believe micro-tiered instruction helps improve the physical health status of students with high or low BMI.	○	○	○		

6. Based on your professional knowledge, please indicate your level of agreement with the following statements. (The following are all single-choice questions. Please select the option that best matches your true feelings by checking "√" from "1=Strongly disagree, 2=Disagree, 3=Neutral/Unsure, 4=Agree, 5=Strongly agree".) [Matrix scale question] *

	1	2	3	4	5
I believe Body Mass Index (BMI) is a key basis for micro-tiered instruction.	○	○	○	○	○
I believe cardiorespiratory endurance level is a key basis for micro-tiered instruction.	○	○	○	○	○
I believe muscular strength level is a key basis for micro-tiered instruction.	○	○	○	○	○
I believe speed quality level is a key basis for micro-tiered instruction.	○	○	○	○	○

	1	2	3	4	5
I believe flexibility level is a key basis for micro-tiered instruction.	○	○	○	○	○
I believe agility level is a key basis for micro-tiered instruction.	○	○	○	○	○
I believe mastered motor skills and proficiency are key indicators for choosing practice difficulty in stratification.	○	○	○	○	○
I believe a micro-stratification scheme should be constructed by integrating BMI + multidimensional physical fitness data + athletic foundation + learning attitude.	○	○	○		

7. Based on the above, which indicators do you think should be used as the core basis for classroom micro-stratification? (Limit to 3 items, rank by importance) [Ranking question, please enter numbers sequentially in the brackets] *

- [ ] Body composition (BMI)
- [ ] Cardiorespiratory endurance
- [ ] Strength quality
- [ ] Speed quality
- [ ] Flexibility quality
- [ ] Agility quality
- [ ] Specific sports skills
- [ ] Technical and tactical application
- [ ] Sports self-confidence

8. What specific suggestions do you have for the implementation of classroom micro-tiered instruction or the modification of this questionnaire? [Fill-in-the-blank question] *

9. Please rate the validity of this questionnaire. (The following are all single-choice questions. Please select the option that best matches your true feelings.) [Matrix scale question] *

	Unreasonable	Somewhat unreasonable	Neutral	Reasonable	Very reasonable
Content validity	○	○	○	○	○
Practical validity	○	○	○	○	○
Construct validity	○	○	○	○	○

110. Basis for answer choices (The following are all single-choice questions. Please select the option that best matches your true feelings.) [Matrix scale question] *

	Small	Medium	Large
Intuitive feeling	○	○	○
Practical experience	○	○	○
References	○	○	○
Theoretical analysis	○	○	○

11. Familiarity with indicators (The following are all single-choice questions. Please select the option that best matches your true feelings.) [Single choice] *

○ Unfamiliar

○ Somewhat unfamiliar

○ Neutral

○ Familiar

○ Very familiar
